# Molecular analysis of three DNA mismatch repair protein variants in Chinese families with suspected Lynch syndrome

**DOI:** 10.3389/fmed.2025.1635964

**Published:** 2025-07-29

**Authors:** Juyi Li, Haichun Ni, Peng Cheng, Yujia Peng, Lei Liu, Xiangyang Wang, Wei Cheng, Hengfei Li, Xiufang Wang, Hongfeng Zhang, Jifa Hu, Aiping Deng, Wei Cai

**Affiliations:** ^1^Department of Pharmacy, The Central Hospital of Wuhan, Tongji Medical College, Huazhong University of Science and Technology, Wuhan, China; ^2^Department of Pathology, The Central Hospital of Wuhan, Tongji Medical College, Huazhong University of Science and Technology, Wuhan, China; ^3^Department of Gastrointestinal Surgery, The Central Hospital of Wuhan, Tongji Medical College, Huazhong University of Science and Technology, Wuhan, China; ^4^Department of Infectious Diseases, Hubei Provincial Hospital of Traditional Chinese Medicine, Wuhan, China; ^5^Department of Pain, The Central Hospital of Wuhan, Tongji Medical College, Huazhong University of Science and Technology, Wuhan, China; ^6^Department of Scientific Research, The Central Hospital of Wuhan, Tongji Medical College, Huazhong University of Science and Technology, Wuhan, China; ^7^Hubei Provincial Engineering Research Center of Intestinal Microecological Diagnostics, Therapeutics, and Clinical Translation, Wuhan, China

**Keywords:** whole exome sequencing, Lynch syndrome, mismatch repair gene, genetic counseling, three-dimensional structure

## Abstract

**Purpose:**

This study aimed to examine pathogenic variations in three families clinically diagnosed with suspected Lynch syndrome (LS).

**Methods:**

Three probands clinically diagnosed suspected LS were subjected to immunohistochemical analysis of DNA mismatch repair (MMR) protein. Whole-exome sequencing and Sanger sequencing were performed to screen pathogenic variations. I-TASSER and PyMOL were used to analyze changes in the functional domains of mutant proteins.

**Results:**

A known missense variation (GRCh37 chr2:g.47702367G>A, MSH2:NM_000251:c.1963G>A:p.V655I), a known stop-gain variant (GRCh37 chr2:g.47709984G>T, MSH2:NM_000251:c.2701G>T:p.E901X), and a known frameshift insertion variation (GRCh37 chr2:g.48032124 dupA, MSH6:NM_000179:c.3514dupA:p.R1172Kfs*5) in Family 1, Family 2, and Family 3, respectively, were observed. The c.1963G>A variation caused the 655th amino acid of MSH2 to change from valine to isoleucine, and there were no significant changes in both the overall and local protein models in MSH2. Further, the c.2701G>T variation caused the 901st amino acid of MSH2 to change from glutamic acid to a premature stop codon in exon 16, and the deletion of amino-acids 901–934 caused changes in the Domain 5 of MSH2 protein. Furthermore, the c.3514dupA variation caused the 1172nd amino acid of MSH6 to change from arginine to lysine, followed by frameshift, which caused changes in the Domain 5 of MSH6 protein.

**Conclusion:**

The missense variation (MSH2:NM_000251:c.1963G>A:p.V655I) and the stop-gain variation (MSH2:NM_000251:c.2701G>T:p.E901X) were considered uncertain significance for LS, and another pathogenic variation (MSH6:NM_000179:c.3514dupA:p.R1172Kfs*5) has been further confirmed.

## Introduction

Colorectal cancer (CRC) is the third most common type of cancer worldwide (1). Lynch syndrome (LS) accounts for 3% patients with CRC and 2% of those with endometrial cancer (EC), and 10–15% of those with DNA mismatch repair (MMR)-deficient tumors (2, 3). LS patients have a high risk of developing CRC (52–82%), EC (40–60%), and several other types of cancer (4).

The LS is caused by germline variants in MMR genes, including *MLH1*, *MSH2* (*EPCAM*), *MSH6*, and *PMS2* accounting for 40–60%, 40–50%, 10–20%, and 2% of LS cases, respectively (5–7). Mutations in the above genes disrupt mismatch repair, which can accelerate the accumulation of somatic mutations and thus the occurrence of tumors (8).

Therefore, it is important to understand the mutation characteristics related to LS, and further conduct genetic counseling based on the results of gene testing, in the Chinese population. In our work, we performed gene sequencing on three families clinically diagnosed with suspected LS and identified three candidate pathogenic variants: a known missense variation (MSH2:NM_000251:c.1963G>A:p.V655I), a known stop-gain variant (MSH2:NM_000251:c.2701G>T:p.E901X), and a known frameshift insertion variant (MSH6:NM_000179:c.3514dupA:p.R1172Kfs*5). Next, we evaluate the spatial impact of candidate pathogenic variants on proteins, and finally, we provide personalized medication guidance to the carriers of these pathogenic variants.

## Methods and materials

### Patients

We obtained written informed consent from the study participants. The Ethics Committee of the Central Hospital of Wuhan approved this study (No. 2020-192). Three probands were clinically diagnosed with suspected LS and underwent partial colectomy or hysterectomy.

### Immunohistochemistry

Tissue samples fixed in formalin and embedded in paraffin were used for pathological detection (hematoxylin-eosin, H&E). Slides were stained with mouse monoclonal antibodies for MLH1, PMS2, MSH2, and MSH6 (9, 10).

### Next generation sequencing

Collect 2 milliliters of peripheral blood from each subject and extract genomic DNA. The Hybridization Capture procedure was performed using the Agilent SureSelect Human All Exon V7 enrichment kit. DNA fragments were sequenced using the NovaSeq^™^ 6000 Sequencing System (Illumina HiSeq 2500 Analyzer, United States) (11, 12). Annotate the genomic variations in this study based on the reference genome UCSC hg19 (9, 13, 14).

### Sanger sequencing

DNA sequencing was performed using ABI 3500 (Thermos, United States). In Family 1, the forward and reverse primers were 5′-CAGGCTATGTAGAACCAATG-3′, 5′-GAGGACTGGCTCAAAGGTAA-3′, respectively; In Family 2, the forward and reverse primers were 5′-GGCAACATAGTGAGACCCTCGT-3′, 5′-TTGATAGCCCATGGGCACTGAC-3′, respectively; In Family 3, the forward and reverse primers were 5′-ATTCTAGGCATCTCAGTAGT-3′, 5′-AAAAGAGAGAGAGACTATGC-3′, respectively.

### Three-dimensional structure

Three-dimensional (3D) structures of MSH2/MSH6 were analyzed and displayed using I-TASSER^1^ and PyMOL^2^ (15, 16).

## Results

### Clinical phenotypes

**Figure 1:**
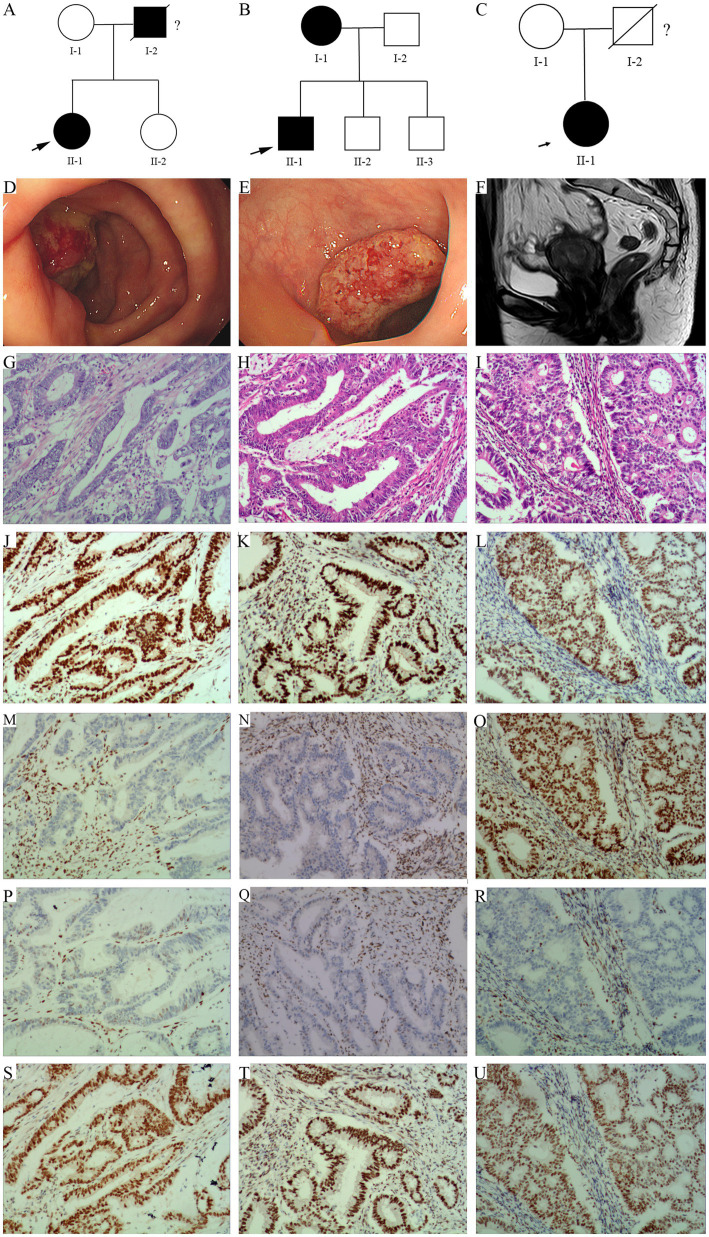
Pedigree structure of family 1, 2, and 3. **(A)** Family 1. **(B)** Family 2. **(C)** Family 3. The arrows indicate the probands. The proband is shown as II-1 in families 1, 2, and 3. Squares are males, circles are females, and crosses indicate deceased individuals. The dark shading represents individuals with LS associated cancer. **(D)** A new tumor in the cecum of the proband in family 1. **(E)** A huge new tumor in the sigmoid colon of the proband in family 2. **(F)** Irregular thickening of the endometrium at the bottom of the uterus, with local clusters protruding toward the uterine cavity in MRI scan in Family 3. **(G–I)** HE staining of the proband’s tumor tissue, **G/H/I** represents Family 1, Family 2, and Family 3, respectively. **(J–U)** Immunohistochemistry. From left to right, the staining of the proband’s tumor tissue from family 1, 2, and 3. From up to down, the antibodies in each line were specific for MLH1, MSH2, MSH6, and PMS2.

In family 1, the proband (II-1, 51 year old female, Figure 1A) underwent right colon surgery after being diagnosed with CRC (51 year old) because of changes in her bowel habits, and her father (I-1) died of cerebral hemorrhage and suspected gastrointestinal cancer. In family 2, the proband (II-1, 51 year old male, Figure 1B) underwent left colon surgery after being diagnosed with colon cancer (51 year old) owing to discomfort in her upper abdomen, and his mother (I-1) was also diagnosed with CRC. In family 3, the proband (II-1, 56 year old female, Figure 1C) underwent a total hysterectomy after being diagnosed with EC (51 year old) following vaginal bleeding. Her father (I-2) had died, but the cause of death was unknown.

Endoscopic examination revealed a huge new tumor in the cecum of the proband of family 1 (Figure 1D), and a huge new tumor in the sigmoid colon of the proband of family 2 (Figure 1E), all with surface ulceration. Magnetic resonance imaging (MRI) of the pelvic cavity revealed irregular thickening of the endometrium at the bottom of the uterus, with local clusters protruding toward the uterine cavity. Diffusion weighted imaging showed diffusion limitation, whereas an enhanced scan showed significant enhancement, involving the muscle layer at the bottom of the uterus of the proband of family 3 (Figure 1F).

### Histological analysis of the tumor tissue

**Figure 2:**
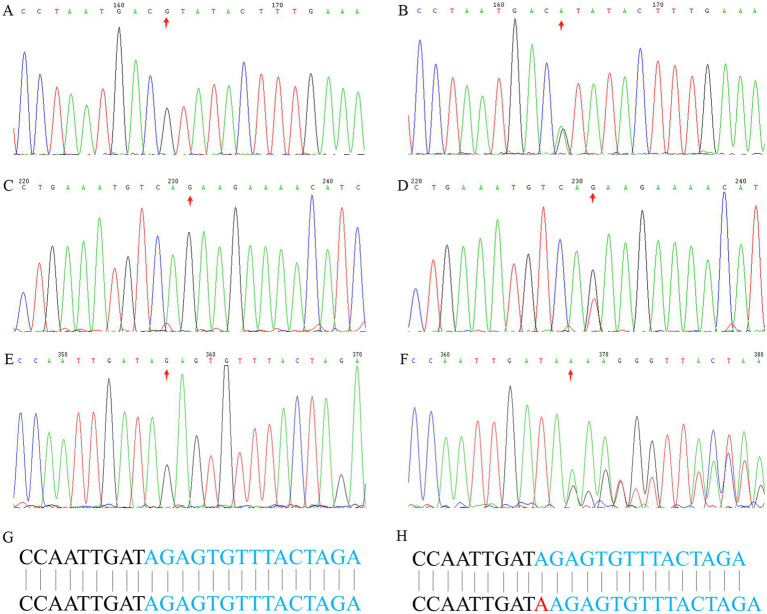
Sanger sequencing analysis. Sanger sequencing of *MSH2* gene (c.1963G>A) of the proband in family 1: **(A)** wild type, **(B)** mutant type. A stop-gain variant (MSH2: c.2701G>T) in Family 2: **(C)** wild type, **(D)** mutant type. A frameshift insertion variant (MSH6: c.3514dupA) in Family 3: **(E)** wild type, **(F)** mutant type, **(G)** wild type base sequence, **(H)** mutant type base sequence.

In family 1 (Figure 1G) and 2 (Figure 1H), the hematoxylin–eosin (HE) staining results showed that the tissue section locally presented an image of mucinous adenocarcinoma, with the cancer penetrating the intrinsic muscle layer and infiltrating into the subserosal fibrous adipose tissue. The proband of family 1 (Figure 1G) manifested with moderately differentiated adenocarcinoma in the right colon, the proband of family 2 (Figure 1H) manifested with moderately to well differentiated adenocarcinoma in the left colon. In family 3, HE staining indicated highly differentiated endometrioid in the uterus of the proband, with cancer cells invading the uterine muscle layer and penetrating to half of its thickness (Figure 1I).

Immunohistochemical staining of the proband’s tumor cells in family 1 and family 2 demonstrated strong positivity for MLH1 (Figures 1J,K) and PMS2 (Figures 1S,T), but no positivity for MSH2 (Figures 1M,N) and MSH6 (Figures 1P,Q) proteins. In family 3, strong positivity for MLH1 (Figure 1L), MSH2 (Figure 1O), and PMS2 (Figure 1U), not for MSH6 (Figure 1R) proteins, was observed.

### Exome and sanger sequencing

We sequenced the exomes of the probands in the three families (Table 1), and average sequencing depth on the target of the probands exceeded 120. A known variant (GRCh37 chr2:g.47702367G>A, MSH2:NM_000251:c.1963G>A:p.V655I) was identified in *MSH2* in family 1, namely rs549467183, and the allele frequency of this variant was 0.0000386 (GnomAD_exomes), 0.0002 (1000G_30X) and 0.000 (East Asian). Multiple statistical methods predicted that the variant will have harmful effects on genes or gene products. The MutationTaster score was 0.987549 and FATHMM score was −1.94, which were defined as deleterious. A known stop-gain variant (GRCh37 chr2:g.47709984G>T, MSH2:NM_000251:c.2701G>T:p.E901X) was identified in *MSH2* in family 2. This variant frequency was not recorded in any database. Multiple statistical methods predicted that the variant will have harmful effects on genes or gene products. The LRT score was 0.000754 and MutationTaster score was 1, which were defined as deleterious. In family 3, a known frameshift insertion variant (GRCh37 chr2:g.48032124 dupA, MSH6:NM_000179:c.3514dupA: p.R1172Kfs*5) was identified in *MSH6*, namely rs63751327. The allele frequency is this variant was 0.0000100 (GnomAD_exomes), 0.00008 (GO-ESP) and 0.00 (East Asian), and the Clinical significance of this variant was defined as pathogenic. The results of Sanger sequencing confirmed the variants (rs549467183, MSH2:NM_000251:c.2701G>T:p.E901X and rs63751327) discovered by whole-exome sequencing in the above mentioned three families (Figure 2).

**Table 1:** Whole-exome sequencing detail of the proband in family 1, 2 and 3.

Sample	Proband in family 1	Proband in family 2	Proband in family 3
Total	71,725,430 (100%)	85,424,840 (100%)	85,177,970 (100%)
Mapped	71,685,396 (99.94%)	85,352,889 (99.92%)	85,128,418 (99.94%)
Properly mapped	71,261,148 (99.35%)	84,714,612 (99.17%)	84,623,102 (99.35%)
Initial_bases_on_target	60,456,963	60,456,963	60,456,963
Total_effective_yield(Mb)	10717.50	12754.43	12715.24
Effective_yield_on_target(Mb)	7320.99	8968.28	8772.81
Average_sequencing_depth_on_target	121.09	148.34	145.11
Bases_covered_on_target	60,103,413	60,276,937	60,148,761
Coverage_of_target_region	99.4%	99.7%	99.5%
Fraction_of_target_covered_with_at_least_100x	50.8%	60.8%	60.0%
Fraction_of_target_covered_with_at_least_50x	78.5%	83.8%	83.8%
Fraction_of_target_covered_with_at_least_20x	93.5%	95.2%	95.2%

### Protein structure prediction

**Figure 3:**
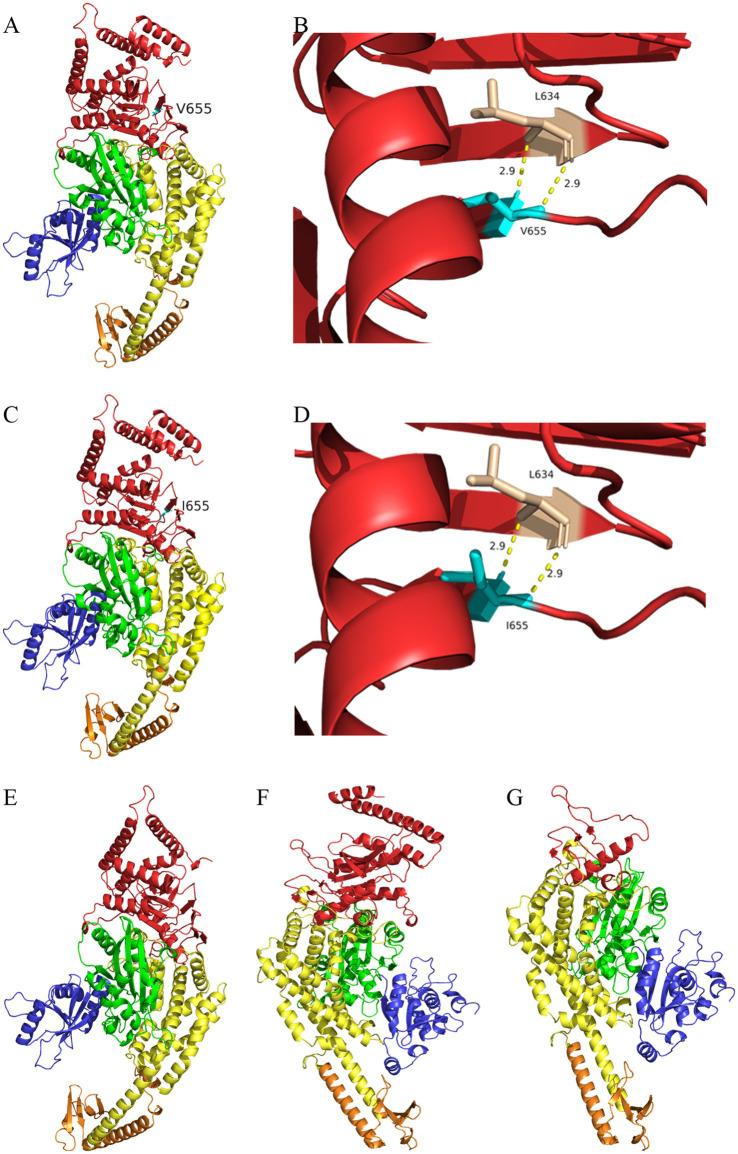
I-TASSER predicts the protein structure of the wild type and mutant of MSH2/MSH6 proteins. **(A)** Three dimensional structure of MSH2 wild-type protein (V655). **(B)** Partial three-dimensional structure of MSH2 wild-type protein (V655). **(C)** Three dimensional structure of MSH2 mutant protein (I655). **(D)** Partial three-dimensional structure of MSH2 mutant protein (I655). **(E)** The deletion of amino acids 901–934 in the mutant MSH2 protein model causing changes in the Domain 5 (red region) region sequence of MSH2 protein. **(F)** Three-dimensional structure of MSH6 wild-type protein. **(G)** The c.3514dupA variant leads to amino acid frameshift, which in turn causes changes in Domain 5 region sequence of MSH6 protein (red region).

In family 1, the c.1963G>A variant caused the 655th amino acid of MSH2 to change from valine (Figure 3A) to isoleucine (Figure 3C). Protein model predictions showed that this variant was located in the β-fold region of the protein, and two hydrogen bonds with L634 were observed at a distance of 2.9 Å before (Figure 3B) and after (Figure 3D) the variant. There were no significant changes in both the overall and local protein models. In family 2, c.2701G>T variant caused the 901st amino acid of MSH2 to change from glutamic acid to a premature stop codon. The deletion of amino acids 901–934 caused changes in the Domain 5 region sequence of MSH2 protein (Figure 3E). In family 3, c.3514dupA variant caused the 1172nd amino acid of MSH6 to change from arginine to lysine, followed by frameshift, causing changes in the Domain 5 region of the MSH6 protein (Figure 3G, wild type of MSH6 shown in Figure 3F).

## Discussion

In this study, the missense variation (MSH2:NM_000251:c.1963G>A:p.V655I) was considered uncertain significance (PP3 + PP4), the stop-gain variant (MSH2:NM_000251:c.2701G>T:p.E901X) in MSH2 was also defined as uncertain significance (PM2 + PP3 + PP4). In addition, the known frameshift insertion variant (MSH6:NM_000179:c.3514dupA:p.R1172Kfs*5) in MSH6 was confirmed as pathogenic.

MMRs play a critical role in DNA replication, genome stability, and mutation avoidance (17–19). Under normal circumstances, strong nuclear staining is a characteristic of MMR proteins, but their loss or decrease in expression indicates a defect in the MMR system (17, 20). LS-related cancers typically exhibit characteristic loss of MMR protein expression, mainly involving MLH1 and PMS2. This loss is usually attributed to germline variations in the MLH1 gene or high methylation of the MLH1 gene promoter. This disrupts its function as a heterodimer of PMS2, resulting in the loss of immunohistochemical expression of MLH1 and PMS2. In contrast, MSH2 gene variants disrupt its function as a heterodimer of MSH6, accompanied by immunohistochemistry deletions of both MSH2 and MSH6 (17, 21, 22).

In family 1, immunohistochemical analysis of MMR proteins suggested double loss of MSH2 and MSH6 expression, and the known variant (MSH2:NM_000251:c.1963G>A:p.V655I) was identified in the proband. The variant was very rare, and the clinical significance of this variant was defined as uncertain significance in ClinVar. This variant was located in the β-fold region of the protein, and showed no significant changes in both the overall and local protein models. However, multiple statistical methods predict that the variant can have harmful effects on genes or gene products. The variant had a FATHMM score of −1.94 and MutationTaster score of 0.987549, and was defined as deleterious. MSH2 mutations account for 36% of patients with the MMR variations (23, 24). The vast majority of variants are nonsense or frameshift mutations, which lead to loss of protein function. However, 18% of MSH2 variants are single base variants, which may cause changes in one amino acid, and the ultimate impact on protein function is often uncertain, therefore, which posing a challenge for doctors and genetic counselors who must manage the disease and determine cancer risk. Therefore, the specific pathogenic mechanism of the missense variant (MSH2:NM_000251:c.1963G>A:p.V655I) needs further research.

The DNA repair system is crucial for repairing errors causing DNA replication. The MSH2-MSH6 protein complex plays an important role in maintaining the mismatch repair mechanism. An interface mutation between the two proteins can impair their function during the repair process (25, 26). In family 1, the missense variation in MSH2 could result in a large number of small fragment deletions or insertions, leading to DNA instability, however, MSH2 forms a dimer with MSH6 genes, ultimately causing double loss of MSH2 and MSH6 expression in the result of immunohistochemical staining. In family 2, the stop-gain variant of MSH2 (NM_000251:c.2701G>T:p.E901X) caused the deletion of amino acids 901–934, leading to changes in the Domain 5 region sequence of MSH2 protein. The nonsense variant in MSH2 gene leaded to the truncation of MSH2 protein or mRNA, resulting in nonsense mediated attenuation, however, MSH2 forms a dimer with MSH6 genes, ultimately causing double loss of MSH2 and MSH6 expression in the result of immunohistochemical staining. In family 3, the known frameshift insertion variant MSH6:NM_000179:c.3514dupA:p.R1172Kfs*5 caused amino acid frameshift, wherein the fifth amino acid encountered a stop codon after frameshift, ultimately resulting in partial deletion of the Domain 5 region sequence of the MSH6 protein. The frameshift variant in MSH6 resulted in the truncation of MSH6 protein or mRNA, leading to nonsense mediated attenuation and ultimately causing loss of MSH6 expression, however, MSH2 expression remained positive, which requires further exploration. Therefore, partial deletion of the structural domain of MSH2 or MSH6 might impair the MSH2-MSH6 complex, thereby impair the activity of the complex and ultimately impair the DNA mismatch repair function of the MSH2-MSH6 complex.

CRCs with deficient MMRs have sustained responses to immune checkpoint inhibitor such as monoclonal antibody against program death 1 (PD-1) (27–29). In 2017, the FDA approved pembrolizumab for the treatment of unresectable or metastatic solid tumors with microsatellite instability-high or MMR defects that have progressed after previous treatment and for adult and pediatric patients without satisfactory alternative treatment options, as well as for the treatment of CRCs with inoperable or metastatic microsatellite instability-high or MMR defects that have progressed well after treatment with fluoropyrimidine, oxaliplatin, and irinotecan (27, 28, 30). Microsatellite instability and MMR defects are interchangeably used as the first pan-cancer biomarkers for the prediction of response to anti-PD-1/PD-L1-therapy (31). Pembrolizumab is the first FDA approved cancer treatment indication based on common biomarkers rather than primary sources (28). In addition, patients with colon cancer who demonstrate microsatellite instability-high or MMR deficiencies have shown improved survival (32). A large amount of preclinical and clinical evidence suggests a possible resistance to 5-FU in these tumors with microsatellite instability-high (33, 34). Therefore, patients with LS will not benefit from fluorouracil treatment, but may be sensitive to PD-1/PD-L1 inhibitors.

At present, less than 10% of individuals undergo genetic testing for CRC in the United States, and the incidence rate of LS is severely underestimated. Furthermore, the proportion of individuals undergoing genetic testing in China is also estimated to be very low. It is recommended that family members with LS undergo genetic counseling and that LS patients or carriers of pathogenic mutations undergo gastroscopy/colonoscopy every 1–2 years (35).

In summary, in this study, the missense variation (MSH2:NM_000251:c.1963G>A:p.V655I) and the stop-gain variation (MSH2:NM_000251:c.2701G>T:p. E901X) were considered uncertain significance for LS, and the pathogenic variation (MSH6:NM_000179:c.3514dupA:p.R1172Kfs*5) was further confirmed. Genetic testing is crucial for the diagnosis and treatment of LS. Finally, patients with LS should not be treated with fluorouracil drugs, and anti-PD1/PD-L1 may be preferred.

## Data Availability

The original contributions presented in the study are publicly available. This data can be found here: NCBI SRA, PRJNA1292369.

## Footnotes

^1^ https://seq2fun.dcmb.med.umich.edu//I-TASSER/

^2^ https://pymol.org/2/
